# Bi-allelic *MYMX* variants cause a syndromic congenital myopathy with recognizable facial palsy, growth restriction, and dysmorphism

**DOI:** 10.1038/s41431-024-01759-9

**Published:** 2024-12-12

**Authors:** Fatima Rahman, Luisa Marsili, Domizia Pasquetti, Aboulfazl Rad, Muhammad Nadeem Anjum, Gabriela Oprea, Huma Arshad Cheema, Barbara Vona, Cesar Augusto Alves, Henry Houlden, Shazia Maqbool, Stephanie Efthymiou, Thomas Smol, Reza Maroofian

**Affiliations:** 1 Department of Developmental-Behavioral Pediatrics, The Children’s Hospital and University of Child Health Sciences, Lahore, Pakistan; 2 University of Lille, ULR7364 RADEME, CHU Lille, Service de Génétique Clinique, Lille, France; 3 Department of Genetics, University Medical Center Utrecht, Utrecht University, Utrecht, Netherlands; 4 Section of Genomic Medicine, Department of Life Sciences and Public Health, Catholic University of Sacred Heart, Rome, Italy; 5 Arcensus Diagnostics, Rostock, Germany; 6 Department of Pediatric Gastroenterology Hepatology and Genetic Diseases, Children’s Hospital and University of Child Health Sciences, Lahore, Pakistan; 7 Institute for Auditory Neuroscience and InnerEarLab, University Medical Center Göttingen, Göttingen, Germany; 8 Institute of Human Genetics, University Medical Center Göttingen, Göttingen, Germany; 9 Division of Neuroradiology, Department of Radiology, Boston Children’s Hospital - BCH Harvard Medical School, Boston, MA USA; 10 Department of Neuromuscular Diseases, UCL Queen Square Institute of Neurology, London, UK

**Keywords:** Genetics research, Neuromuscular disease, Disease genetics

## Abstract

Myogenic fusion, primarily regulated by the Myomaker and Myomixer proteins, is essential for skeletal muscle development, yet its mechanisms remain poorly understood. This study presents the clinical and molecular details of the third and fourth reported patients with biallelic variants in *MYMX*, the gene that encodes Myomixer. We identified a homozygous truncating variant [c.107 T > A (p.Leu36Ter)] and a homozygous stop-codon loss variant [c.255 A > G (p.Ter85TrpextTer41)] in *MYMX*, both associated with a complex neuromuscular syndrome characterized by generalized hypotonia, congenital myopathy, facial nerve palsy, growth restriction and facial dysmorphism. Additional variable features include hearing loss (confirmed in one patient, suspected in the other), scoliosis, joint contractures, cleft palate, hypoglossia, potentially contributing to Pierre Robin sequence, and abnormalities on neuroimaging studies including cerebellar atrophy and Chiari 1 deformity. Comparative analysis of patients with pathogenic variants in *MYMK* and *MYMX*, including our cases, reveals largely overlapping phenotypes, underscoring their synergistic role in myofiber formation and implicating their involvement in the etiology of neuromuscular conditions.

## Introduction

During the physiological process of skeletal muscle development and regeneration, the fusion of mononucleated myoblasts to form multinucleated myofibers plays a pivotal role; nevertheless, in contrast to earlier stages of myogenesis, the mechanism and regulation of myogenic fusion are poorly understood. Studies have proven that two muscle-specific regulators, namely Myomaker [1] (coded by *MYMK*) and Myomixer [2] (coded by *MYMX*) act together as a molecular switch of myoblast fusion during muscle formation [3].

Despite numerous evidence supporting the biological role of Myomaker and Myomixer, their correlation with human diseases is largely unknown; recently, biallelic variants in *MYMK* were shown to cause a rare congenital neuromuscular disorder, known as Carey-Fineman-Ziter syndrome (CFZS1; OMIM #254940). CFZS1, clinically described in nearly 20 patients [4], is characterized by hypotonia, developmental delay, Pierre Robin and Moebius sequences. In subsequent studies, *MYMK* homozygous or compound heterozygous variants were identified in 9 patients from 5 unrelated families [5] and in a 69-year-old man, presenting with juvenile-onset proximal myopathy [6].

Consequently, a homozygous nonsense variant in *MYMX* was identified in two sibs with facial weakness and myopathy [7]. Experimental evidence, including defective fusion of patient-derived myoblasts and the premature death of mouse models bearing the *MYMX* variant, suggests that biallelic *MYMX* pathogenic variants cause a monogenic human disease (CFZS2, OMIM #619941), highly overlapping with Carey-Fineman-Ziter syndrome [8–10].

In this study, we detail the clinical and molecular characteristics of two unrelated patients, both carrying novel homozygous variants in *MYMX* and presenting with complex neuromuscular symptoms. Additionally, we compare our clinical findings to those of patients with similar pathogenic variants in *MYMK* and those clinically diagnosed with CFZS1, providing insights into their phenotypic similarities and distinctions.

## Results

Patient 1, a six-year-old boy and the second child of consanguineous Pakistani parents, was born following a pregnancy complicated by polyhydramnios and delivered via caesarean section due to breech presentation. His family history includes a miscarriage of pregnancy, complicated by severe fetal abnormalities. Birth parameters were as follows: Length: 48 cm (50th percentile), Weight: 2900 g (3rd percentile), Head circumference: 35 cm (34th percentile). At birth, he suffered from failure to thrive and low weight, necessitating hospitalization. Early infancy revealed hypotonia, and developmental delays were evident as he walked without support at 28 months and spoke his first words at 2.5 years. By age six, he showed a vocabulary of nearly 100 words, although his intelligence quotient was borderline. Behaviour was described as mildly aggressive.

Neurological examinations indicated muscle atrophy and reduced bulk in all limbs, hypotonia, bilateral flexion contractures and down-going plantar responses. He experienced numbness and tingling in hands and feet, and a partially positive Gowers sign. Contractures prevented elicitation of deep tendon reflexes. Visual assessments noted amblyopia and reduced visual acuity accompanied by bilateral strabismus. The disease course was non-progressive.

**Fig. 1:**
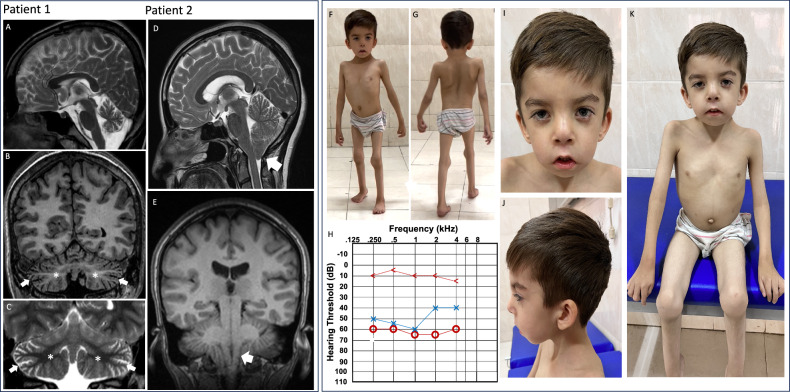
Clinical features of patients. Left panel: **A**–**C** Sagittal and coronal T2 weighted imaging (WI), and coronal T1-WI brain MRI imagines from proband 1, showing selective atrophy of the cerebellum including the vermis and cerebellar hemispheres (arrow, **B**, **C**) and abnormal signal changes affecting the dentate nuclei (asterisks, **B**, **C**). **D**, **E** Sagittal T2-WI and coronal T1-WI brain MRI imagines from proband 2 showing pointed cerebellar tonsils slightly displaced caudally resulting effacement of the CSF columns through the craniocervical junction (borderline Chiari type 1 deformity). Right panel: **F**, **G** Stills extracted from a walking video depicting proband 1’s gait. **H** Pure-tone audiogram for the proband 1, showing elevated air conduction thresholds in dB hearing level for the right (red circles) and left ears (blue crosses). Bone conduction thresholds for the right ear are indicated by ‘<‘. **I**–**K** Clinical photographs of the proband 1.

Brain MRI at two years old revealed selective cerebellar atrophy, including the vermis and hemispheres, with abnormal signal changes in the dentate nuclei (Fig. 1A–C). Serum concentrations of ammonia and lactate were within the normal range. A cardiology review showed no abnormalities. He also presented with a cleft palate, surgically repaired at age of 2 years, an underdeveloped scrotum with bilateral cryptorchidism, talipes equinovarus, severe scoliosis, ulnar deviation of the fingers, and brachydactyly. At 4.9 years, he was diagnosed with moderate conductive asymmetric hearing loss (2 and 4 kHz) (Fig. 1H). Notable facial dysmorphism was also observed, including microcephaly, plagiocephaly, Pierre Robin and Mobius-Robin sequence, anteverted nares, epicanthic folds, ptosis, short nose, thin vermillion border, long philtrum, down-slanting eyes (Fig. 1I–K). At six years old, his growth parameters were significantly restricted [Weight: 10 kg (−4 SD), Stature: 95 cm (−4 SD)].

**Fig. 2:**
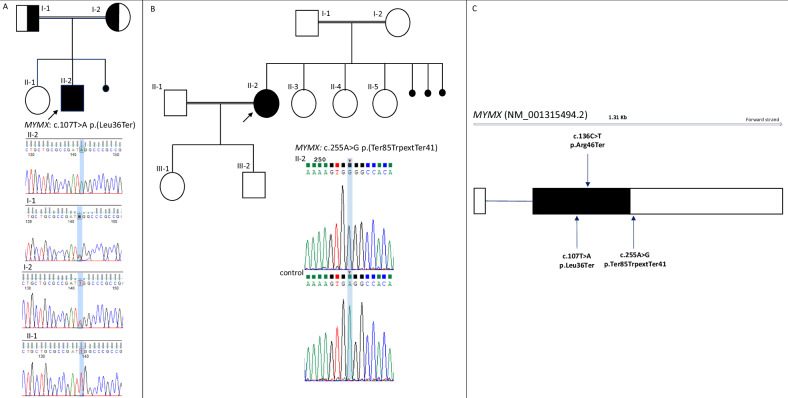
Families pedigree and human *MYMX* structure. **A** Patient 1 pedigree and Sanger sequencing electropherograms displaying segregation of the *MYMX* (NM_001315494.2) c.107 T > A (p.Leu36Ter) variant. Key: II-2: proband; I-1: father; I-2: mother; II-1: healthy sister. **B** Family 2 pedigree and Sanger sequencing electropherograms displaying the *MYMX* (NM_001315494.2) c.255 A > G p.(Ter85TrpextTer41) variant. **C** Gene structure of human MYMX (NM_001315494.2*)*: Boxes represent exons; white boxes denote untranslated regions, and black box indicates open reading frame. *MYMX* variant from previously reported patients is shown in the upper part, and *MYMX* variants identified in our patients are shown in the lower part.

Trio-exome sequencing revealed a novel homozygous truncating c.107 T > A (p.Leu36Ter) variant in *MYMX* (NM_001315494.2) in the proband. This variant was inherited from healthy carriers parents (Fig. 2A). Additionally, whole genome analysis of the proband ruled out the presence of copy number variations (CNVs) or genomic rearrangements. The WES methodology is described in the Supplementary Materials.

Patient 2, a 30-year-old woman, is the first of four daughters of healthy consanguineous parents of French, Senegalese, Mauritanian and Libanese ancestry, with no relevant family history. She had two uncomplicated pregnancies and gave birth to two unaffected children (Fig. 2B). She was diagnosed with congenital bilateral facial nerve diplegia at birth and presented with feeding difficulties during infancy, but no additional information about perinatal period or early childhood was available. Velopharyngoplasty for submucous cleft soft palate was performed at age 9 years. Since the age of 18 years, she experienced chronic headache and episodes of bilateral upper extremities weakness and pain. She was referred to the clinical genetics department because of syndromic congenital facial diplegia. Physical examination revealed short stature [147 cm (−3 SD), Weight 49 kg (−0.5 SD), Fronto-occipital head circumference 52.3 cm (−2 DS)], hand muscle atrophy, and craniofacial dysmorphisms including retrognathia, strabismus, and posteriorly rotated ears with large ear lobes. Hypernasal speech was also noted. The neurological examination showed bilateral facial diplegia without any other cranial nerve involvement, mild distal muscle weakness in all limbs, weak deep tendon reflexes in the upper limbs, in the absence of cerebellar or sensory impairment. She had normal intellectual capacity.

Electromyography was performed at 19 years. Bilateral facial nerve conduction was abnormal. The right facial nerve exhibited a latency of 4.6 ms (range: 3.4–4.0 ms) and an amplitude of 0.6 mV (range: 200–500 mV), while the left had a latency of 4.3 ms and an amplitude of 0.4 mV. The motor nerve conduction study of the right median, right and left external sciatic-popliteal, and left internal sciatic-popliteal was within reference ranges, showing latencies ranging from 2.2 to 4.1 ms, and conduction velocities between 52 and 68 m/s. The sensory nerve conduction study of the right sural, left sural, and right median nerves, showed normal amplitudes (range 98–167 µV) and velocities (range 45–53 m/s). Brain MRI was conducted at age 18 years, revealing bordeline Chiari type 1 malformation, excluding other abnormalities in the central nervous system (Fig. 1D, E). Surgical decompression was performed at the age of 36 because of severe chronic headache. Histopathological analysis of the resected cerebellar tonsils did not reveal any pathological findings. Cardiac assessment was normal. Hearing loss was suspected on a subjective basis, but the patient was not able to undergo audiological testing.

Karyotype analysis performed during childhood was normal (46, XX). CGH array revealed a 400 kb heterozygous interstitial duplication of chromosome 5p13.2p13.3, which contained no OMIM-morbid gene and was classified as variant of unknown significance. Finally, solo exome sequencing identified the homozygous c.255 A > G p.(Ter85TrpextTer41) variant in the *MYMX* (NM_001315494.2) gene, subsequently confirmed by Sanger sequencing. No other likely pathogenic or pathogenic variant was identified. Parental samples were not available for segregation analysis. The WES methodology for patient 2 is described in the Supplementary Materials.

## Discussion

The *MYMX* c.107 T > A (p.Leu36Ter) variant identified in patient 1 introduces a premature stop codon at position 36 of the Myomixer protein, leading to a loss of its main extramembrane portion, which includes the critical AxLyCxL motif. This motif is essential for protein function and it is highly conserved across species. Interestingly, another reported variant in *MYMX* (p.Arg46Ter) results in a similarly truncated protein that also lacks the AxLyCxL motif. *MYMX* c.107 T > A (p.Leu36Ter) variant identified in patient 1 is a novel variant, absent from both ClinVar and population databases; conservation scores suggest a partially conserved residue at amino acidic codon 36 (PhyloP100Way: 3.865) and it is classified as *pathogenic according to ACMG guidelines* (Table S3) [11]. Given the variant’s location and the consistent phenotype observed in our patient, it is plausible that the c.107 T > A variant may result in a similar molecular effect, though functional validation is necessary.

The *MYMX* c.255 A > G p.(Ter85TrpextTer41) identified in patient 2 is expected to affect the translation termination (stop) codon and cause a 41-amino acid extension at the extramembrane C-terminal end of the Myomixer protein. This novel variant, absent from ClinVar database, is observed in gnomAD v4 (https://gnomad.broadinstitute.org/, accessed on 07/06/2024), with an allele frequency of 0.00002053 (allele count = 1) and it is classified as likely pathogenic (Table S3). Although functional validations are required, it is possible to speculate on different pathomechanisms for the p.Ter85TrpextTer41 stop-loss variant, including modifications to the C-terminal organization. Specifically, biological evidence suggests that the ectodomains of MYMX, which include the organization of alfa helix-1 and alfa helix-2, are critical for the development of the fusion pore by inducing membrane stress. Alterations to the alpha-helix structure could potentially disrupt the positive curvature necessary for fusion pore formation [12]. Another possibility includes the loss of a potential protective effect of the terminal amino acid on the extracellular AxLyCxL motif.

Conversely, causative variants for CFZS1 syndrome are typically missense variants located in the intramembrane domains of the Myomaker protein, which functions biologically alongside Myomixer. These findings support the hypothesis that Myomaker and Myomixer proteins act collaboratively in a stepwise reaction essential for membrane fusion.

Despite the limited number of reported cases, our observations suggest that pathogenic variants in *MYMK* and *MYMX* share a highly overlapping core phenotype (Table S2). This phenotype includes motor developmental delay, facial weakness, hypotonia, growth restriction, feeding difficulties, and velopharyngeal insufficiency. Notably, IQ scores across all patients remain above the threshold for intellectual disability. Among the facial dysmorphisms, retro/micrognathia is prevalent, often associated with cleft palate and hypoglossia, contributing to the Pierre Robin sequence in half of the cases. Additionally, nonspecific dysmorphisms such as down-slanting palpebral fissures, epicanthal folds, ptosis, and a broad-tipped upturned nose are common, although these features are not exclusive to this condition. Hearing loss was confirmed in patient 1 and suspected but not confirmed in patient 2, suggesting that this feature may be more common than previously reported. Similarly, scoliosis and joint contractures are present in a variable percentage of patients, 34.5% and 71.4%, respectively.

The core phenotype is consistent even in CFZS1 patients lacking molecular confirmation. Nonetheless, the possibility of other pathogenic variants in different disease-causing genes in this patient’s group cannot be discounted.

## Supplementary information

Supplemental material 1

Supplemental material 2

## Data Availability

All data relevant to the study are included in the article.
