# Supplementary material for: Bi-allelic MYMX variants cause a syndromic congenital myopathy with recognizable facial palsy, growth restriction, and dysmorphism
Source: Eur J Hum Genet. 2024 Dec 12;33(4):552–5. doi: 10.1038/s41431-024-01759-9 (PMC11986149; doi:10.1038/s41431-024-01759-9)
Supplement: Supplementary file 1 — Supplemental material 1 [file 41431_2024_1759_MOESM1_ESM.docx]

**Table S1**

| **Our patients** | **Classic CFZS** ^(5,6)^ | **CFZS-like** ^(7)^ |
| --- | --- | --- |
|  |  |  |
| ***MYMX***:c.107T>A (p.Leu36Ter) | ***MYMK***: c.2T>A (1) | ***MYMX***: c.136C>T p.(Arg46Ter) (2 patients, both homozygous) |
| ***MYMX***: c.255A>G p.(Ter85TrpextTer41) | ***MYMK***: c.271C>A p.Pro91Thr |  |
|  | ***MYMK***: c.298G>A p.Gly100Ser |  |
|  | ***MYMK***: c.461T>C p.Ile154Thr (1 patient, homozygous) |  |
|  | ***MYMK***: c.553T>C p.Cys185Arg |  |

**Legend:** Summary of previously reported variant in classic CFZS and CFZS-like patients.

**Table S2**

|  | **Our patients** | ***Patients with MYMX PV*** (7) | **Patients with *MYMK* PV** (5,6) | **CFZS1 clinical diagnosis** (4) |
| --- | --- | --- | --- | --- |
| *Retro/micrognathia* | 2/2 | 2/2 | 9/9 | 18/18 |
| *Pierre Robin sequence* | 2/2 | NA | 3/9 | 8/9 |
| *Moebius sequence* | 1/2 | NA | NA | 16/18 |
| *Down-slanting palpebral fissures* | 1/2 | 2/2 | 4/9 | 12/14 |
| *Epicanthal folds* | 1/2 | 0/2 | 8/9 | 10/15 |
| *Ptosis* | 1/2 | 0/2 | 8/9 | 11/17 |
| *Upturned nose* | 1/2 | 1/2 | 9/9 | 16/16 |
| *Broad nasal tip* | 1/2 | NA | 9/9 | 12/16 |
| Microcephaly | 2/2 | 1/2 | NA | 4/18 |
| Cleft palate/velopharyngeal insufficiency | 2/2 | 1/2 | 6/9 | 12/18 |
| Feeding difficulties | 2/2 | 1/2 | 7/9 | 14/18 |
| Joint contractures | 1/2 | 0/2 | 8/9 | 11/15 |
| Scoliosis | 1/2 | 2/2 | 5/9 | 2/15 |
| Facial muscle weakness | 2/2 | 2/2 | 9/9 | 18/18 |
| Hypotonia | 2/2 | 2/2 | 9/9 | 18/19 |
| Delayed motor development | 1/1 | 1/2 | 9/9 | 17/17 |
| Intellectual disability | 0/2 | 0/2 | 0/9 | 0/6 |
| Growth delay | 2/2 | 1/2 | 7/9 | 2/7 |
| MRI abnormalities | 2/2 | NA | 0/4 | 6/11 |
| Hearing loss | 1/1 | 0/2 | 1/9 | 1/18 |

**Legend:** Prevalence of CFZS main clinical features among patients with Pathogenic Variants in *MYMK* and *MYMX*, and clinically diagnosed CFZS1 patients lacking molecular confirmation. Facial dysmorphisms are highlighted in italics. 'Na' indicates data not available. (PV) pathogenic variants.

**Table S3**

|  | *MYMX* c.107T>A variant in **proband 1** | *MYMX* c.255A>G variant in **proband 2** |
| --- | --- | --- |
| ACMG guidelines criteria (11) | **PVS1:** Null variant in a gene where loss of function (LOF)  is a known mechanism of disease | **PM4:**Protein length changes due to in-frame deletions/insertions in a non-repeat region or stop-loss variants |
|  | **PM1:**  Located in a mutational hot spot and/or critical and well-established functional domain (e.g. active site of an enzyme) without benign variation | **PM2 :**Absent from controls (or at extremely low frequency if recessive) in Exome Sequencing Project, 1000 Genomes or ExAC |
|  | **PM2:**Absent from controls (or at extremely low frequency if recessive) in Exome Sequencing Project, 1000 Genomes or ExAC | **PP3:**Multiple lines of computational evidence support a deleterious effect on the gene or gene product |
|  | **PP3 :**Multiple lines of computational evidence support a deleterious effect on the gene or gene product | **PP4:**Patient’s phenotype or family history is highly specific for a disease with a single genetic etiology |
|  | **PP4:**Patient’s phenotype or family history is highly specific for a disease with a single genetic etiology |  |
| ACMG guidelines classification | **Pathogenic (class V)** | **Likely pathogenic (class IV)** |

**Legend:** ACMG guidelines criteria and variant classifications for *MYMX* variants in patient 1 and patient 2.
