# Supplementary material for: Bi-allelic MYMX variants cause a syndromic congenital myopathy with recognizable facial palsy, growth restriction, and dysmorphism
Source: Eur J Hum Genet. 2024 Dec 12;33(4):552–5. doi: 10.1038/s41431-024-01759-9 (PMC11986149; doi:10.1038/s41431-024-01759-9)
Supplement: Supplementary file 2 — Supplemental material 2 [file 41431_2024_1759_MOESM2_ESM.docx]

**Material and Metods**

**Patient 1**

Genomic DNA was extracted from peripheral blood samples using standard phenol-chloroform procedures. Whole-exome sequencing (WES) was conducted on each proband, by Macrogen, Korea. Briefly, target enrichment was performed using 2 μg of genomic DNA and the SureSelectXT Human All Exon Kit version 6 (Agilent Technologies, Santa Clara, CA, USA) to create barcoded WES libraries. These libraries were sequenced on the HiSeqX platform (Illumina, San Diego, CA, USA) with a coverage depth of 50x. Quality control of the sequencing reads was performed using FastQC (http://www.bioinformatics.bbsrc.ac.uk/projects/fastqc).

The bioinformatics filtering strategy focused on exonic and donor/acceptor splicing variants. Based on the pedigree and phenotype, priority was given to rare variants (<0.01% in public databases, such as the 1000 Genomes Project, NHLBI Exome Variant Server, Complete Genomics 69, and Exome Aggregation Consortium [ExAC v0.2]) that fit a recessive (homozygous or compound heterozygous) or de novo inheritance model, as well as variants in genes previously associated with developmental delay, intellectual disability, and other neurological disorders.

**Patient 2**

Samples were prepared using Illumina DNA Prep Enrichment (Illumina, San Diego, US-CA). A 150 bp paired-end exome sequencing was performed on an Illumina NovaSeq 6000 platform, with an average on-target coverage of 100X and analyzed using GATK best practices. Reads were aligned to the Genome Reference Consortium human genome build 38 (GRCh38), and the variants were annotated using Variant Effect Predictor in combination with the in-house pipeline ANATOLE2. The identified variants were filtered based on their frequency, quality, and functional impact. Variants with a minor allele frequency greater than 1% in public databases (gnomAD) were excluded. Variants located in genes known to be associated with the patient’s phenotype according to the HPO database were given higher priority. Selected variants were confirmed by Sanger sequencing in the affected probands and their parents.
